# Anæsthesia

**Published:** 1868-01

**Authors:** J. Hardman

**Affiliations:** Muscatine, Iowa


### ANÆSTHESIA.

BY J. HARDMAN, MUSCATINE, IOWA.

Read before the Iowa State Dental Association at Lyons, July 12, 1867.

ANÆSTHESIA is usually defined "a privation of sensation." And it may be either general or local, its influence pervading the whole system, or but a part. This definition is a little too general for the Dentist's purpose, as it would imply the absence of sensibility without reference to cause. My remarks therefore will have reference more exclusively to the insensibility induced by artificial agents introduced, either into the general system or applied to a part.

First then, we would call attention to the agents used to induce general insensibility; and of these we wish to notice only three, viz.: chloroform, ether and nitrous oxyd, or protoxyd of nitrogen. The history of these, both as to their chemical discovery and their first application for anæsthesia, I will not attempt in this paper; but will take it for granted that each one present, like myself, feels the greater interest in their effects and relative merits.

A very important quality in each of these three agents for the induction of anæsthesia is, that they should be *pure* and free from all agents of a deleterious character. And as Dentists are seldom furnished with the means by which to thoroughly test this fact, it becomes frequently a delicate question of conscience how far we should indulge in the use of these agents, if we have reason to infer constitutional unfitness in any of our patients.

The apparent physical effects of these three agents are very similar; and it is often remarked by patients who have used each that the sensations have been quite similar. Yet, it is evident that ether and chloroform produce this effect in a very different way from nitrous oxyd. Leaving the *modus operandi* of each to yourselves, I will state it as my conviction that the effect being similar, a relative state of the system is brought about; only differing in proportion to intensity of agent, relative quantity used, manner of exhibition and peculiarity of subject.

Chloroform is very prompt, and if inhaled freely will, in from one to five minutes produce insensibility, and even in most cases unconsciousness. If the patient is healthy the pulse varies but little from the normal beat,—at first a little accelerated and full, but soon as insensibility becomes somewhat general it is reduced in volume and frequency. An irregular or intermittent pulse indicates danger, and is cause for alarm.

This agent is in its effects so powerful, subtle, and consequently in many cases so dangerous, that the operator in Dentistry who makes a general use of it for anæsthesia at all, cannot be regarded as blameless; and is indeed morally reckless of the health and lives of his patients. The many cases of fatality that have taken place in the hands of careful and scientific men, and the destitution of any reliable antidote, admonish us to *beware.*

Sul. Ether (well washed) is my favorite for the induction of general anæsthesia; and, for fear of making my subject tiresome, I will at once refer to my favorite mode of procedure in its exhibition. I ascertain the idiosyncrasies, hereditary transmissions and present health of patient by inquiry, —examine the pulse, place the ear over the heart, (and for this purpose have at hand a good plain stethescope,) ascertain when the last meal was taken, and its quality and quantity; avoid a full, late meal; avoid cases where much organic disease of the lungs exists, where any evident organic disease of the heart or brain; and in most cases of gestation. Always have a third person present, especially if the patient is a female. Examine the mouth, decide what forceps will be needed, and place them in easy reach. Decide upon a particular tooth to commence with; place napkins, water, etc., in reach; put the chair and patient in proper position. Advise the patient how to take large and full inspirations; admonish him to breathe fully every time and to continue so long as he has power. Use small glass inhaler with sponge, with aperture for a free supply of air. Have the patient hold the nose tightly with the fingers of the left hand, place the inhaler to the mouth at each inspiration, but remove at each expiration. Frequently examine the pulse, watch the countenance; and when the hand of patient drops from the nose, proceed to operate. In this I take up no time with lancet; but crowd well up on the necks and roots of the teeth with the points of the forceps, and extract as rapidly as possible. As soon as done, or if patient recovers sensibility, bring the head forward over the spittoon to prevent too much blood passing into the fauces. If desired to remove still more teeth, in a few moments repeat the inspiration of ether. When done, wet the head with cold water and order cup of strong coffee, which usually remove the effects of ether in a short time.

If ether is inhaled by full, deep and free inspirations and expirations, its effect is quickly sedative, pleasant, sufficiently anæsthetic, its effects pass off quickly, and the necessary quantity used, small. When administered slowly, little by little, it intoxicates and stimulates highly; requires a large quantity taken; promotes unpleasant delirium and its effects leave the patient tardily.

Protoxyd of Nitrogen is now the popular general anæsthetic among the Dentists of this country. It has been but a few years since its introduction to any extent in the profession, yet many thousands have successfully used it for the annihilation of pain in the Dental chair. I have not had much experience with this agent, and will but give my opinion principally in reference to its comparative merits as it seems to me. Its effects are too transient, and much more so than ether, is more troublesome to keep ready; and disagrees with most cases of lung complaints.

A CASE to illustrate: Mrs. B., aged 40, sanguino-bilious temperament, wished to inhale gas, having had attacks of hæmoptisis. I discouraged her intentions; but offered her the inhaler to test the effect, to her own mind as well as myself, by two inspirations. This produced excessive cough, with a general tightness of the chest as being bound by a cord; these symptoms continuing for many minutes,—patient declaring that gas would suffocate her. Two days after I gave her ether without the least hack of a cough, and in 20 minutes after declared herself all right and left the office.

The gas she tried was evidently pure; used it direct from the gas-holder; had never *been* inspired; and others had taken of the same with pleasant effect. Two or three similar cases have occurred in my charge; and when using it, I feel more need of extra caution than in the use of ether.

*Local Anæsthesia.*—For this purpose there have been quite a number of agents presented to the profession at various periods. Most of them consisting of some combination of powerful narcotics, such as chloroform with tincture of aconite, or chloroform with tincture of opium, etc. These were recommended to be applied to the gums surrounding the tooth. Also, the influence of galvanism conveyed to the tooth by making the forceps a part of the galvanic circle from a battery. Then also came congelation of the tooth and gums by means of freezing mixtures, as a combination of ice and salt applied by variously constructed devices. Among all these nothing practicably reliable being established until the *congelation* by means of an atomized spray; which is of so much anæsthetic power and so useful that no Dentist of operative practice should be without an instrument. I have had the benefit of one now for nearly a year, and can assure my Dental brethren I would hardly know how to do without it.

It is *true*, that for extraction it will not do for universal use; as where the sudden reduction of the temperature excites or aggravates pain, especially in neuralgic cases. It is also impossible to establish its influence to purpose about the molars of the lower jaw. Yet a large proportion of cases exist where it can be used with facility, and is so completely anæsthetic that patients feel highly delighted with it; will sit for ten or a dozen extractions, where they experience no more pain than is usual for one in the ordinary way. But this is not the extent of its use; the obtunding of sensitive dentine can be so completely and readily accomplished, and still leave the tooth and nerve healthy, that here it is invaluable. And by it, in many cases the exposed nerve can be frozen to insensibility and at once extracted; making the successful treatment of the tooth much more certain and speedy. Again, in the treatment of alveolar abscesses it will sustain an enviable reputation. My mode is to congeal by applying spray to the gums opposite to the diseased root; and then quickly with stiff narrow chisel or sharp drill cut freely to the seat of the disease, cut up and remove the suppurating membrane; and then medicate with escharotics and alteratives. I use the Bigalo instrument with direct spray. Have used Richardson's instrument, but without satisfaction. It is evident that much improvement in instruments and in the application of this benumbing process can be accomplished, and the field for its usefulness much enlarged.

Gentlemen, I have now briefly and imperfectly given my ideas and preferences in regard to anæsthesia in Dentistry; I wish to add but a few more thoughts, and these upon the moral justification of the Dentist in the use of those powerful *general* agents. Feeling that the history of fatality connected with them, and the evident nature of the action these agents have upon the nervous centres, involves a great responsibility and interest, not only in those who take them, but more emphatically in those who encourage their use.

The intelligent operator must not shut his eyes to the anatomical structure and the functional nature of animal life. He sees that the brain is the great vital centre; and that this *vital principle* holds sacred influence and connection over every part of the system, by a beautifully constructed system of nerves. These nerves all centre, either directly to the *brain* or its appendages. Nerves supplying sensation and motion to the head, and many parts of the body passing off directly from the encephalon, while many leave from the medulla oblongata and medulla spinalis, and supply the various organs and muscles of the body and extremities.

For our purpose we may refer alone to the peculiar nerve power which creates or exerts muscular motion. This is of two kinds, *voluntary* and *involuntary;* the latter of these deserves our especial consideration. The *heart*, the lungs and the secreting organs are almost exclusively supplied by this variety of nerve influence, and the great principal seat of this influence is, that part of the base of the brain called the medulla oblongata. From this the nerves emerge that supply the heart, that great engine of *involuntary* muscular action, and upon whose constant and normal operation without which life is not sustained for a moment.

If then, an agent is introduced into the general system that has the power to suspend the higher functions of the brain, and thus cut off *voluntary* action, causing the muscles of the head, neck, body and limbs to fall placid, insensible and lifeless; I need not ask, what can be the condition of the whole system, suppose the agent exerts its influence upon the base of the brain, the *medulla oblongata?* Can any one be so wise and so expert that he may give chloroform to the annihilation of life in that portion of the cerebrum from which the trifacial nerve originates, and leave unparalyzed the medulla oblongata? It has been no doubt correctly stated by M. Flourens, that all the other parts of the brain may be safely suspended of their forces, provided the medulla oblongata be unattacked by the agent, and so long as it retains its energy and remains free from the anæsthetic agent, it is capable of calling the other portions into life and activity through its own force. Hence, he calls medulla oblongata, the "*vital tie.*" But may not this *vital tie*, this *golden cord* be as liable to become paralyzed as any other part? And if it be, then death is the result! I have no doubt in my own mind that this is the mode of operation in 99-100ths of the fatal cases from these tremendous agents.

Of the three agents I have above referred to, I consider chloroform the most dangerous, from its known activity and the many cases of death it has caused. I had the misfortune to be present at the last breath of one of its victims, and I crave not another opportunity.

*Nitrous oxide* is next dangerous in my estimation, judging from its activity and evident mode of operation. And it presents not a very clean history; no better than that of chloroform at its age: and we may well look for misfortunes to occur from the reckless use many make of it. I regard *ether* the most safe of all three, and for support to this conclusion would mainly refer to the long and almost limitless use made of it, and not a well authenticated case of fatality upon record as yet. I have also by experiments upon animals, tested the relative powers of chloroform and ether. Have kept cats, dogs and rats under the influence of ether for hours, and still they recovered perfectly. But the same animals have in several cases succumbed in two to five minutes under chloroform.

The principal *post mortem* features of these cases were exhibited in the head and thorax. The brain being pale, blanched or quite deficient of blood; while the lungs, the large vessels and heart were a mass of sanguinous engorgement. I think conclusively showing the utter destruction of that nervous force of involuntary contractile power.

Gentlemen, to annihilate pain and the horrors of an operation, when done without recklessness is benificent; but, as highly as the art of mitigating pain has become, we are still groping very much in the dark; and let it not be forgotten that the life of a fellow being is of too much estimate to slight the moral bearing of this question; and I therefore ask you to join your efforts to help perfect *safe*, as well as *efficient* anæsthesia.

MUSCATINE, Iowa, June 27, 1867.

